# Resilience to height loss of articular cartilage of osteoarthritic stifle joints of old pigs, compared with healthy cartilage from young pigs in a tribological pin—on—plate exposure, revealing similar friction forces

**DOI:** 10.1371/journal.pone.0250244

**Published:** 2021-04-23

**Authors:** Jan P. Engelhardt, Andy Schütte, Svetlana Hetjens, Gregor Reisig, Markus L. Schwarz

**Affiliations:** 1 Department of Experimental Orthopedics and Trauma Surgery, Medical Faculty Mannheim of the University of Heidelberg, Mannheim, Germany; 2 Department of Medical Statistics, University Medicine Mannheim, Medical Faculty Mannheim of the University of Heidelberg, Mannheim, Germany; University of Memphis, UNITED STATES

## Abstract

**Introduction:**

We saw a lack of data on the biomechanical behavior of degenerated articular cartilage (OA) compared with that of healthy cartilage, even though the susceptibility to wear and tear of articular cartilage plays a key role in the progression of osteoarthritis (OA). Therefore, we performed a comparison between naturally occurring OA and healthy cartilage from pigs, before and after tribological stress.

**Aim:**

The aim of the study was to compare OA-cartilage with healthy cartilage and to analyze the resilience to tribological shear stress, which will be measured as height loss (HL), and to friction forces of the cartilage layers. The findings will be substantiated in macro- and microscopical evaluations before and after tribological exposure.

**Methods:**

We assessed stifle joints of fifteen old and sixteen young pigs from the local abattoir radiologically, macroscopically and histologically to determine possible OA alterations. We put pins from the femoral part of the joints and plates from the corresponding tibial plateaus in a pin-on-plate tribometer under stress for about two hours with about 1108 reciprocating cycles under a pressure of approximately 1 MPa. As a surrogate criterion of wear and tear, the HL was recorded in the tribometer. The heights of the cartilage layers measured before and after the tribological exposure were compared histologically. The condition of the cartilage before and after the tribological exposure was analyzed both macroscopically with an adapted ICRS score and microscopically according to Little et al. (2010). We assessed the friction forces acting between the surfaces of the cartilage pair–specimens.

**Results:**

Articular cartilage taken from old pigs showed significant degenerative changes compared to that taken from the young animals. The macroscopic and microscopic scores showed strong alterations of the cartilage after the tribological exposure. There was a noticeable HL of the cartilage specimens after the first 100 to 300 cycles. The HL after tribological exposure was lower in the group of the old animals with 0.52 mm ± 0.23 mm than in the group of the young animals with 0.86 mm ± 0.26 mm (p < 0.0001). The data for the HL was validated by the histological height measurements with 0.50 mm ± 0.82 mm for the old and 0.79 mm ±0.53 mm for the young animals (p = 0.133). The friction forces measured at the cartilage of the old animals were 2.25 N ± 1.15 N and 1.89 N ± 1.45 N of the young animals (p = 0.3225).

**Conclusion:**

Unlike articular cartilage from young pigs, articular cartilage from old pigs showed OA alterations. Tribological shear stress exposure revealed that OA cartilage showed less HL than healthy articular cartilage. Tribological stress exposure in a pin–on–plate tribometer seemed to be an appropriate way to analyze the mechanical stability of articular cartilage, and the applied protocol could reveal weaknesses of the assessed cartilage tissue. Friction and HL seemed to be independent parameters when degenerated and healthy articular cartilage were assessed under tribological exposure in a pin–on- plate tribometer.

## Introduction

Diarthrodial joints are covered with a thin layer of articular cartilage. The joints allow free movement of the bones, and the articular cartilage tissue provides support and enables the distribution of joint loading while serving as a low friction bearing surface [1, 2]. Apart from those main functions, articular cartilage sustains loads which can amount to up to 18 MPa [3, 4] while suffering minimal wear [5]. The preservation of these mechanical properties is crucial in order to avoid wear [6]. However, loss or degenerative processes of articular cartilage in the context of trauma or in combination with degenerative pathologies may alter those biomechanical properties, including the susceptibility to wear [7, 8]. Height loss (HL) is a main parameter when performing indentation and creep measurements of viscoelastic tissue like articular cartilage [9]. Basalo et al. determined the frictional response of chondroitinase ABC treated and untreated articular cartilage [10]. They recorded the creep displacement response as creep strain normalized to the thickness of the cartilage specimens [10].

HL of the cartilage layers of a joint is referred to as “narrowing of joint space” for the radiological diagnosis of osteoarthritis (OA) according to Kellgren and Lawrence [11, 12].

OA is a frequent pathology of the articular cartilage tissue changes, affecting millions of patients worldwide [7, 13, 14]. Accepted criteria used to describe OA changes are: the Kellgren & Lawrence (K&L) score [11] for the evaluation of radiographic findings, the International Repair Society score for macroscopic changes [15] and the Mankin–score [16] for microscopic scoring. Under *in vitro* conditions, the investigation of cartilage tissue loss due to tribological phenomena in healthy cartilage has been widely investigated [6, 17–24]. But we found there was a lack of data describing the comparison between the biomechanical behavior of articular cartilage with degenerative changes predominantly found in older individuals and the healthy cartilage of young individuals. In the context of osteoarthritic cartilage, most studies used artificial degradation of cartilage, e.g. by surgical intervention [25–27], enzymatic degradation [28–30], mechanical abrasion [31, 32] and gene knockout studies [33] as a means to simulate OA processes. However, literature contrasting healthy cartilage tissue to spontaneously progressed OA-cartilage is rare, particularly in the context of mechanical wear and tear.

Some animal species, like the pig, show degenerative changes of articular cartilage in older animals [34, 35]. Hennerbichler et al. found that the articular cartilage of stifle joints of skeletally mature pigs aged 2 to 3 years developed degenerative changes comparable with those of human OA [34]. Our working group recently identified the local slaughterhouse as a source for degenerately changed menisci in the stifle joints of pigs aged approx. 5 years [36].

Wear of cartilage is defined as abrasion, adhesion and surface fatigue [17] and can be investigated using several qualitative and quantitative methods. These include the biochemical analysis of cartilage [24, 37, 38], changes in cartilage surface roughness [5, 24, 39, 40] and the characterization of altered cartilage layer thickness [5, 26, 41, 42].

Processes of wear and tear under physiological conditions are still poorly understood and are therefore an important field of research in order to address the difficulties in OA research.

In this context, tribological exposure seemed to be an appropriate method to analyze the mechanical properties [43, 44] and the resilience of articular cartilage.

Shear forces are induced by tribological exposure in terms of relative movements of surfaces and may stress the cartilage surface and layers depending on the duration of the stress and the condition of the tissue [5, 45–48]. Shear stress can be quantified using the measurements of the friction forces acting at the surface of the cartilage tissue surface in a pin–on–plate tribometer [5]. The HL of the cartilage layer under tribological exposure could indicate the resilience and stability of the cartilage in terms of degenerative or other changes [5]. Shear stress can be accurately applied in a pin—on—plate tribometer on the cartilage surface under reproducible conditions *in vitro*, even if the test conditions do not exactly reflect the physiological situation. Thus, a pin–on–plate tribometer could be used to determine wear and tear regarding the biomechanical properties of the cartilage tissue and the fatigue characteristics.

The purpose of the study was [1] to explore the hypothesis that cartilage from elderly porcine tibiofemoral joints was significantly affected by spontaneous OA-alterations compared to the cartilage of young joints. If this was the case and significant differences were detected, it would then be possible to explore the second hypothesis [2], namely that the height loss (HL) of the cartilage samples, serving as surrogate indicator of wear and tear regarding the stability of the tissue, would be significantly different after tribological exposure where the friction forces representing the shear stresses in both groups are to be assessed. [3] Effects were to be evaluated macro- and microscopically in both groups after exposure.

## Material and methods

### Specimens and preparation

Within 12 hours after commercial slaughter, 31 porcine stifle joints (*sus scrofa domestica; Schwäbisch-Hällisches-Landschwein*) with a fully intact joint capsule were obtained from a local abattoir (Fleischversorgungszentrum (FVZ) Mannheim GmbH, Mannheim, Germany) and dissected for further processing. Of these 31 joints, 15 were obtained from approximately 5 years old pigs and 16 from approximately 6 months old pigs, resulting in two different groups: old and young.

7 joints from the left and 8 joints from the right knee were obtained from the old pigs, which were all females, and from the young pigs, we harvested 6 joints from the left and 10 joints from the right knee. All stifle joints were randomly collected and were picked up after the animals had been disassembled.

In order to expose the articular cartilage of the distal femur as well as the proximal tibia for macroscopic evaluation regarding OA, the joints were dissected and soft tissue along with ligaments and menisci as well as the patellae were removed. Blood and any other contaminants were rinsed off, using phosphate buffered saline (PBS).

Osteochondral cylindrical plugs with a diameter of 5 mm were punched out of the medial and lateral femur condyles, using instruments specially designed for this purpose as described in a previous study [49]. The plugs were extracted from the most affected area, and in case there were no macroscopically visible degenerative changes, from the main loading zone. These plugs served as pins in the pin–on—plate device (see below). A second plug was harvested next to the first one, serving as a control specimen for histological evaluation in terms of OA and cartilage height.

**Fig 1:**
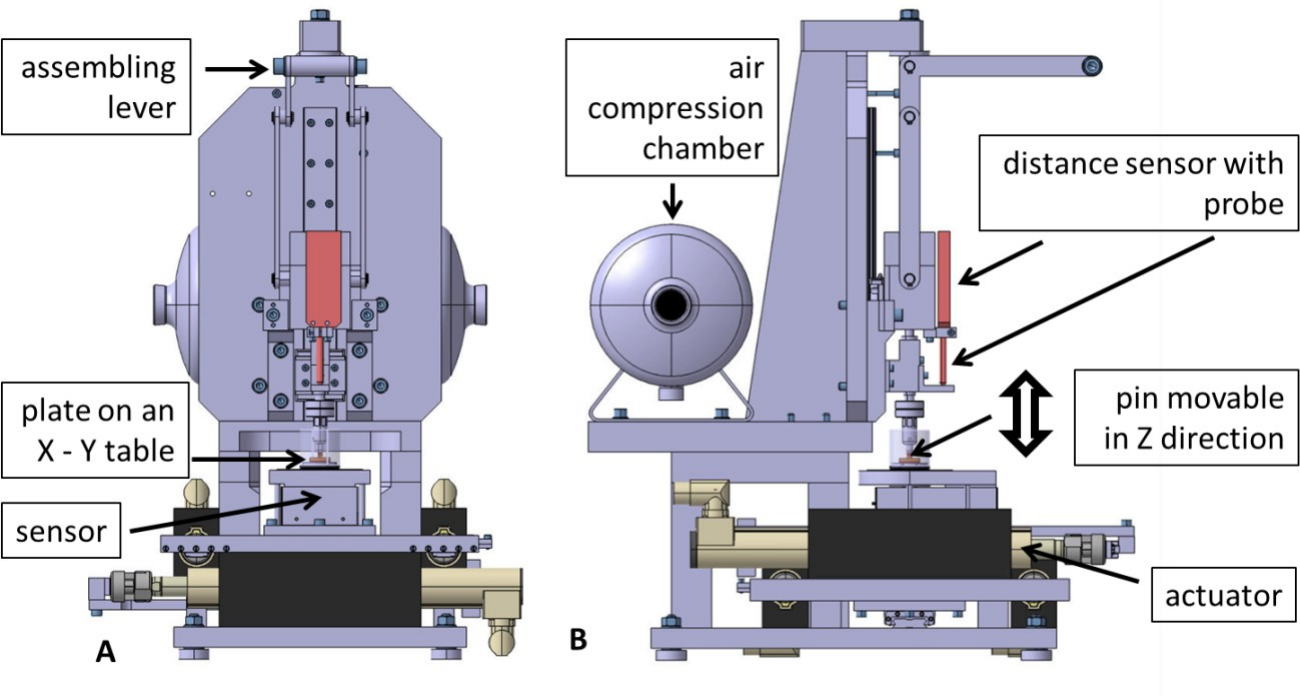
Technical drawing of front (A) and side view (B) of the tribometer in testing mode. The normal force was applied through air pressure on the osteochondral plate via the movable osteochondral pin. The plate was moved on an X–Y table, driven by linear actuators. Height displacements were measured via the distance sensor over the reciprocating trajectories. The experiments were performed in PBS as lubricant. Tubes and cables are not shown.

Osteochondral plates (20 x 20 mm) where taken from the medial and the lateral tibia plateau, using a square punch. During the tribological examinations these served as counterparts of the corresponding femoral pins. The subchondral bone facilitated the fixation in the specimen mount of the pin-on-plate device (Fig 1) [22].

The plate specimens from the tibia plateaus were used for both the histological analyses and the mechanical examinations. The harvesting processes were carefully performed without touching the surfaces, thus avoiding any damage of the cartilage layer. The specimens were stored in PBS in order to prevent them from dehydrating.

Each joint yielded two pairs of tibiofemoral specimens, one from the medial and one from the lateral compartment. Thus, we assessed the tribological exposure on 124 specimens in total, 60 from old (15 medial and 15 lateral pairs) and 64 from young (16 medial and 16 lateral pairs) porcine stifle joints.

### Assessment of osteoarthritis

#### Radiographic

Before we prepared the stifle joints, they were x-rayed in an X-ray imaging cabinet in anterior-posterior as well as in lateral view (Faxitron X-Ray Corporation, Buffalo Grove, USA). The focal distance was approximately 60 cm and the tube voltage 50 kV over an exposure time of 5 seconds. The images where then assessed according to the Kellgren and Lawrence score [11].

#### Macroscopic

**Fig 2:**
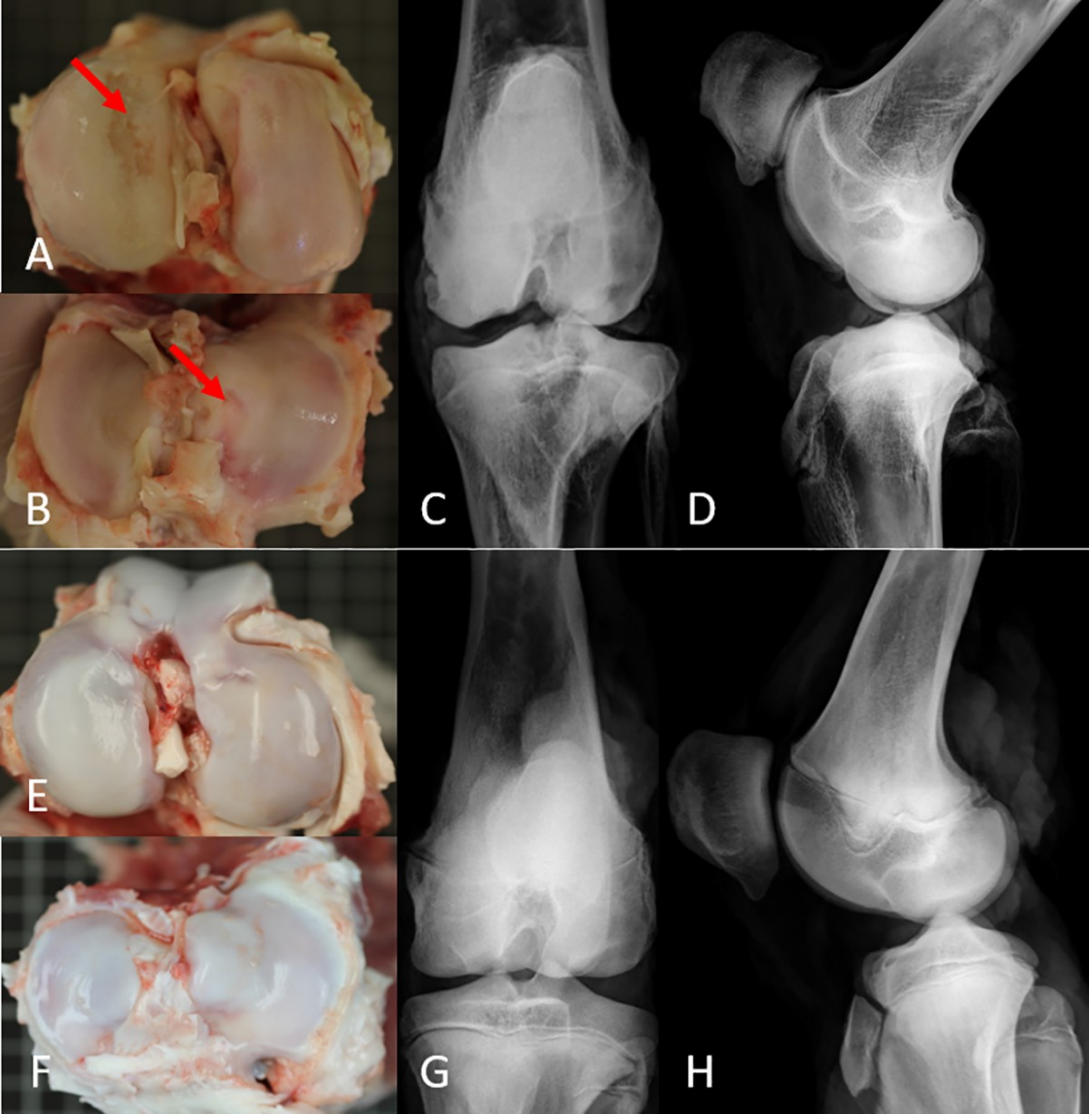
View of the exposed stifle joints of pigs and corresponding X-rays. Femoral condyles (A) and the tibial plateau (B) of a stifle joint originating from an old pig show severe macroscopically visible OA alterations (red arrows); deep articular cartilage loss led to the exposure of the subchondral bone. (C) and (D) show the X- rays of the joint in the anterior–posterior aspect (C) and in the side view (D). Images (E) and (F) show the stifle joint originating from a young pig. Only slight alterations on the medial tibia plateau (F) can be detected. (G) and (H) show the X-rays of the same joint. The growth plates indicate the young age of the animal.

After we had exposed the joints (Fig 2) we assessed the cartilage visually to determine osteoarthritic changes according to the International Cartilage Repair Society System (ICRS) [15] and classified them after having numerically adapted the score (Table 1). The grading was performed, taking into consideration each topographical region of the joints (medial/lateral, femoral/tibial) from which the pins and the plates were harvested. Images of the cartilage surfaces were taken with a camera (Canon EOS 7D, Canon Macro Lens EF 100 mm 1:2.8, Canon Inc., Tokyo, Japan) and digitally saved.

**Table 1:** Numerical adaption of the ICRS cartilage classification system according to Brittberg and Winalski [15]. The numerical adaptation was necessary for clearer statistical analyses.

ICRS Grade	numerical adaptation	macroscopical alterations on cartilage
**0**	0	normal
**1 A**	1	intact surface but fibrillation
**1 B**	1,5	superficial lacerations and fissures
**2**	2	defects < 50% of the cartilage thickness
**3 A**	3	defects > 50% of the cartilage thickness
**3 B**	3,25	defects reaching to the calcified layer
**3 C**	3,5	defects extending to the subchondral bone plate
**3 D**	3,75	formation of blisters
**4A**	4	defects extending to the subchondral bone
**4 B**	4,5	defects extending deep into the subchondral bone

#### Microscopic

For the microscopic assessment, the control pins were fixated in 4% formaldehyde (J. T. Baker / Avantor Performance Materials, Center Valley, USA) immediately after harvesting. The mechanically tested pins and plates were fixed immediately at the end of the tribological exposure. The plates were cut into 2–3 mm thick coronal osteochondral slices including the entire width of the plate specimen. The pins were processed as a whole with a cut running parallel to the axis of the cylindrical pin. After washing the samples that were fixed in formaldehyde, they underwent a decalcification process using EDTA (Merck, Darmstadt, Germany) for 21 days. EDTA was then washed out and the specimens were automatically dehydrated (TP1020 Leica, Wetzlar, Germany). After embedding them in paraffin using a casting implement (EG1140C Leica, Wetzlar, Germany), 5 μm thick slices were cut and mounted on glass slides (R. Langenbrick, Emmendingen, Germany). Before the staining procedure, the slides were deparaffinized with xylene and rehydrated in a series of water and ethanol blends. Staining was performed using toluidine blue (10 min, 0.4% (w/v) toluidine blue in 0.1 M sodium acetate pH 4.0; Merck, Darmstadt, Germany) together with a Fast-green counter stain (3 min, 0.02% (w/v); Merck, Darmstadt, Germany) according to Getzy et al. and Little et al. [50, 51]. The slides were then dehydrated in a series of alcoholic solution with an ascending alcoholic concentration, and finally conserved by Eukitt (O. Kindler GmbH, Freiburg, Germany).

The histologic analyses were performed using a light microscope (DMRE Leica, Wetzlar, Germany) with a mounted camera (DFC 300 FX Leica, Wetzlar, Germany), and photographs were taken.

According to the recommendations of the Osteoarthritis Research Society International (OARSI) histopathology initiative, the assessment was performed using the Little et al. scoring system [51].

### Testing device and tribological exposure protocol

The femoral pins and the corresponding tibial plates were combined to pair—specimens and mounted in a tribological testing device with the pin as upper and fixed part and the plate as lower and moving part (Fig 1) [22]. 15 medial and 15 lateral compartments from the old pigs and 16 medial and 16 lateral compartments from the young animals were included in the tribological exposures.

The pair—specimens underwent tribological exposure under reciprocating motion in the pin-on-plate tribometer, as had been previously described in detail [22]. Compressed air in a pneumatic cylinder was used to apply a constant contact pressure of approximately 1 MPa thus allowing to compensate for irregularities of the cartilage surface of the tibial plate. The vertical load applied over the pin on the plate was measured using a three-axis force sensor (K3D60 10N/10N/50N, ME-Meßsysteme, Hennigsdorf, Germany). The tribological exposures were conducted at room temperature (20°C ± 2°C) with a sliding velocity of 4 mm/s [5, 17, 29, 40, 52] over a distance of 13 mm moving in the horizontal plane. The exposure consisted of 1108 reciprocating cycles, leading to a running time of 2.05 h [40]. We used PBS as a lubricant while keeping the tibio—femoral pair—specimens immersed in the solution during the examination.

**Fig 3:**
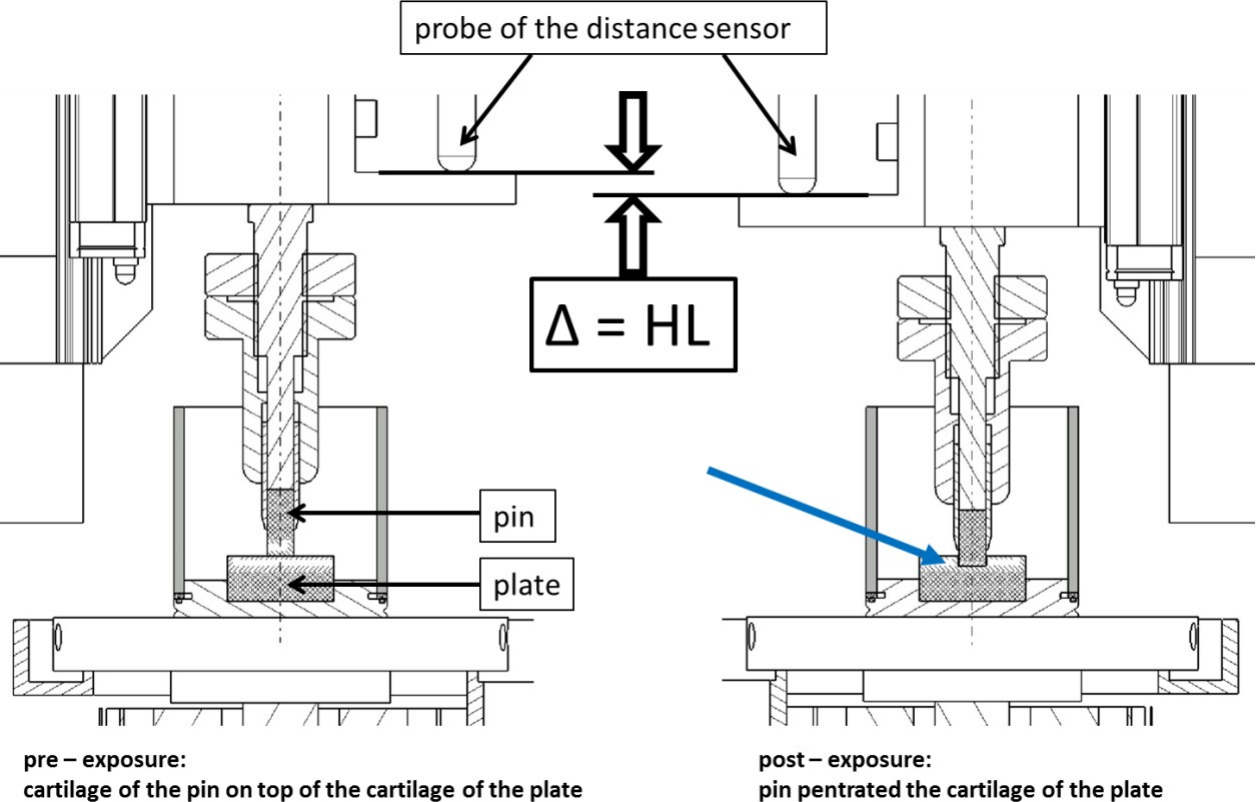
Technical sketch of how the height loss (Δ = HL) of the cartilage layers was measured under tribological exposure of the osteochondral pin and plate. It was measured with the distance sensor shown in Fig 1. The blue arrow indicates the pin penetrating the surface of the plate. Note: both cartilage layers lost height.

The vertical displacement of the pin was continuously monitored using a software (Labview, National Instruments, Austin, USA) that processed the data delivered by a distance sensor (DT32P, Sony, Japan) with an accuracy of ± 5 μm at a rate of 1 kHz. The HL of the pair–specimens and the applied load were observed over the entire running time and recorded over a distance of 3 mm in the very middle of the path. The HL was calculated in a vertical direction, measuring the increasing difference between the initial value at the first cycle and the following cycles in millimeters (Fig 3).

F_x_ represents the forces acting horizontally in the direction of the movement of the plate. They were provided by the three–forces sensor. The friction force for each reciprocating cycle was determined by taking the mean of the measured forces F_x_ in both directions, forwards and backwards.

### Microscopic evaluation after the tribological exposure

After the tribological exposure, pins and plates were treated for histological analyses as described above. We analyzed both the control pins and the worn ones, as well as the intact and the affected areas of the plates using the scoring system described by Little et al. [51]. Thus, we were able to distinguish between alterations of the cartilage which existed before and those which were induced by the tribological exposure.

### Microscopic measurement of the height of cartilage before and after tribological exposure

The height of the cartilage layers of the osteochondral specimens (pins and plates) were measured digitally, using the photographs of the histological slides (Preview, Apple Inc., USA). The scale bar on each photograph served as a reference point when measuring the cartilage thickness in mm, using a digital ruler. The cartilage height before tribological exposure was measured at the control pins and at the intact areas of the plate. The cartilage height after tribological exposure was measured at the worn pins and the affected areas of the plates.

### Stratification of the cartilage from old animals regarding OA

To find an answer to the question if the age of the animal or the expression of the OA could influence the HL, we selected specimens from the group of the old animals and created two sub–groups based on their grade of OA. With regard to the compartments, we set a threshold at ≤ 8.5 scoring points and another threshold at ≥ 11.5 scoring points, according to the Little et al. scoring system (Little et al., 2010). The group of specimens with the lower grading was labeled “OA—“, and the group of specimens with the higher grading was labeled “OA +”. We averaged the scoring points of the femoral and the tibial specimens to achieve representative data for the assessed compartments.

### Statistical analysis

The various score values were assessed according to the compartment the specimens were taken from: medial or lateral and femoral and tibial. We averaged the respective femoral and tibial data (OA scores), representing the compartment.

For the descriptive statistics, we calculated the mean and standard deviation (Excel 2019, Microsoft, Redmond, USA) and performed box and whiskers plots (Origin 8.6.0.G, OriginLab Corporation, Northampton, USA).

The Wilcoxon-Mann-Whitney-Test (Wilcoxon rank-sum test) was used to investigate differences between the two groups: old and young.

The t-test procedure was applied to analyze the HL difference between the old and the young group.

The measured values of the forces acting horizontally (F_x_) showed positive and negative values. For the evaluation of the friction forces, the negative values were changed to positive values so that the friction forces were presented as absolute values. The t–test procedure was used to investigate differences of the friction forces between the groups: old and young, and the compartments of the stifle joints, medial or lateral. The correlation between HL and friction force F_x_ was investigated using the Pearson correlation coefficient.

The statistical analyses were performed using the software SAS 9.3 (SAS, Heidelberg, Germany).

The level of significance was set as 0.05.

## Results

### Assessment of the osteochondral specimens before tribological exposure

#### Radiological changes in terms of OA

We found a significant difference (*p* = 0.0002) in the radiographic score according to Kellgren and Lawrence [11] between the 16 stifle joints originating from the old animals (1.87 ± 0.99) and 15 stifle joints from young (0.19 ± 0.54) (Table 2).

**Table 2:** The table provides a synopsis of the data of degenerative changes (points), heights and HL in the cartilage of the stifle joints from old and young pigs before and after tribological exposure and the friction forces. For the medial and lateral compartments of the stifle joints the HL was calculated using the tribometer and the histologically measured changes of the cartilage heights. The radiological assessments were only made on the complete joints before the tribological exposure. “n”specifies the number of assessed specimens.

		old (5 years) animals			young (6 month) animals		
parameter	anatomical localisation	n	before tribological exposure; mean ± SD; (95% confidence interval)	after tribological exposure (mean ± SD); (95% confidence interval)	n	before tribological exposure (mean ± SD); (95% confidence interval)	after tribological exposure (mean ± SD); (95% confidence interval)
**radiological assessment**	** **	** **	grade	grade		grade	grade
score acc. Kellgren / Lawrence 1957	joint	15	1.87 ± 0.99 (95% CI: 1.32–2.42)		16	0.19 ± 0.54 (95% CI: -0.10–0.48)	
**macroscopical assessment**			points	points		points	points
ICRS score acc. Brittberg et al. 2003	medial	30	1.97 ± 0.91 (95% CI: 1.63–2.31)	2.73 ± 0.91 (95% CI: 2.39–3.07)	32	0.30 ± 0.49 (95% CI: 0.12–0.48)	1.78 ± 1.35 (95% CI: 1.29–2.27)
	lateral	30	1.41 ± 0.47 (95% CI: 1.23–1.59)	2.08 ± 0.78 (95% CI: 1.79–2.37)	32	0.16 ± 0.37 (95% CI: 0.03–0.29)	2.20 ± 1.44 (95% CI: 1.68–2.72)
	all specimens	60	1.69 ± 0.77 (95% CI: 1.49–1.89)	2.41 ± 0.90 (95% CI: 2.18–2.64)	64	0.23 ± 0.44 (95% CI: 0.12–0.34)	1.99 ± 1.40 (95% CI: 1.64–2.34)
**histological assesment**			points	points		points	points
score acc. Little et al. 2010	medial	30	11.07 ± 3.52 (95% CI: 9.76–12.38)	13.57 ± 5.33 (95% CI: 11.58–15.56)	32	4.72 ± 1.85 (95% CI: 4.05–5.39)	8.56 ± 5.78 (95% CI: 6.48–10.64)
	lateral	30	9.50 ± 3.09 (95% CI: 8.35–10.65)	11.13 ± 4.52 (95% CI: 9.44–12.82)	32	5.38 ± 2.37 (95% CI: 4.53–6.23)	9.50 ± 5.65 (95% CI: 7.46–11.54)
	all specimens	60	10.28 ± 3.38 (95% CI: 9.41–11.15)	12.35 ± 5.06 (95% CI: 11.04–13.66)	64	5.05 ± 2.12 (95% CI: 4.52–5.58)	9.03 ± 5.68 (95% CI: 7.61–10.45)
**height histological slides**			height [mm]	height [mm]		height [mm]	height [mm]
	pin medial before tribology	15	1.36 ± 0.59 (95% CI: 1.03–1.69)		13	1.79 ± 0.47 (95% CI: 1.51–2.07)	
	pin medial after tribology	15		1.01 ± 0.55 (95% CI: 0.71–1.31)	14		1.28 ± 0.40 (95% CI: 1.05–1.51)
	pin lateral before tribology	15	0.90 ± 0.25 (95% CI: 0.76–1.04)		14	1.16 ± 0.19 (95% CI: 1.05–1.27)	
	pin lateral after tribology	15		0.86 ± 0.26 (95% CI: 0.72–1.00)	13		0.93 ± 0.16 (95% CI: 0.83–1.03)
	plate medial before tribology	15	1.31 ± 0.42 (95% CI: 1.08–1.54)		13	0.98 ± 0.26 (95% CI: 0.82–1.14)	
	plate medial after tribology	14		0.88 ± 0.32 (95% CI: 0.70–1.06)	13		0.59 ± 0.41 (95% CI: 0.34–0.84)
	plate lateral before tribology	15	1.06 ± 0.36 (95% CI: 0.86–1.26)		14	0.88 ± 0.24 (95% CI: 0.74–1.02)	
	plate lateral after tribology	15		0.86 ± 0.54 (95% CI: 0.56–1.16)	14		0.37 ± 0.13 (95% CI: 0.29–0.45)
**height loss histological**			height loss [mm]			height loss [mm]	
	medial (pin+plate before tribology—pin+plate after tribology)	14	0.80 ± 0.88 (95% CI: 0.29–1.31)		13	0.86 ± 0.69 (95% CI: 0.44–1.28)	
	lateral (pin+plate before tribology—pin+plate after tribology)	15	0.23 ± 0.69 (95% CI: -0.15–0.61)		13	0.71 ± 0.30 (95% CI: 0.53–0.89)	
	all specimens	29	0.50 ± 0.82 (95% CI: 0.20–0.82)		26	0.79 ± 0.53 (95% CI: 0.58–1.00)	
**height loss in the tribometer**			height loss [mm]			height loss [mm]	
	medial	14	0.63 ± 0.25 (95% CI: 0.49–0.77)		11	0.92 ± 0.31 (95% CI: 0.71–1.13)	
	lateral	15	0.42 ± 0.16 (95% CI: 0.33–0.51)		13	0.81 ± 0.20 (95% CI: 0.70–0.94)	
	all specimens	29	0.52 ± 0.23 (95% CI: 0.44–0.61)		24	0.86 ± 0.26 (95% CI: 0.75–0.97)	
**friction forces**			force [N]			force [N]	
	medial	13	2.40 ± 0.92 (95% CL: 1.85–2.96)		11	1.26 + 0.98 (95% CI: 0.61–1.92)	
	lateral	15	2.12 ± 1.34 (95% CL: 1.38–2.86)		13	2.42 + 1.61 (95% CI: 1.45–3.40)	
	all specimens	28	2.25 ± 1.15 (95% CL: 1.81–2.70)		24	1.89 + 1.45 (95% CI: 1.28–2.50)	

#### Macroscopic

**Fig 4:**
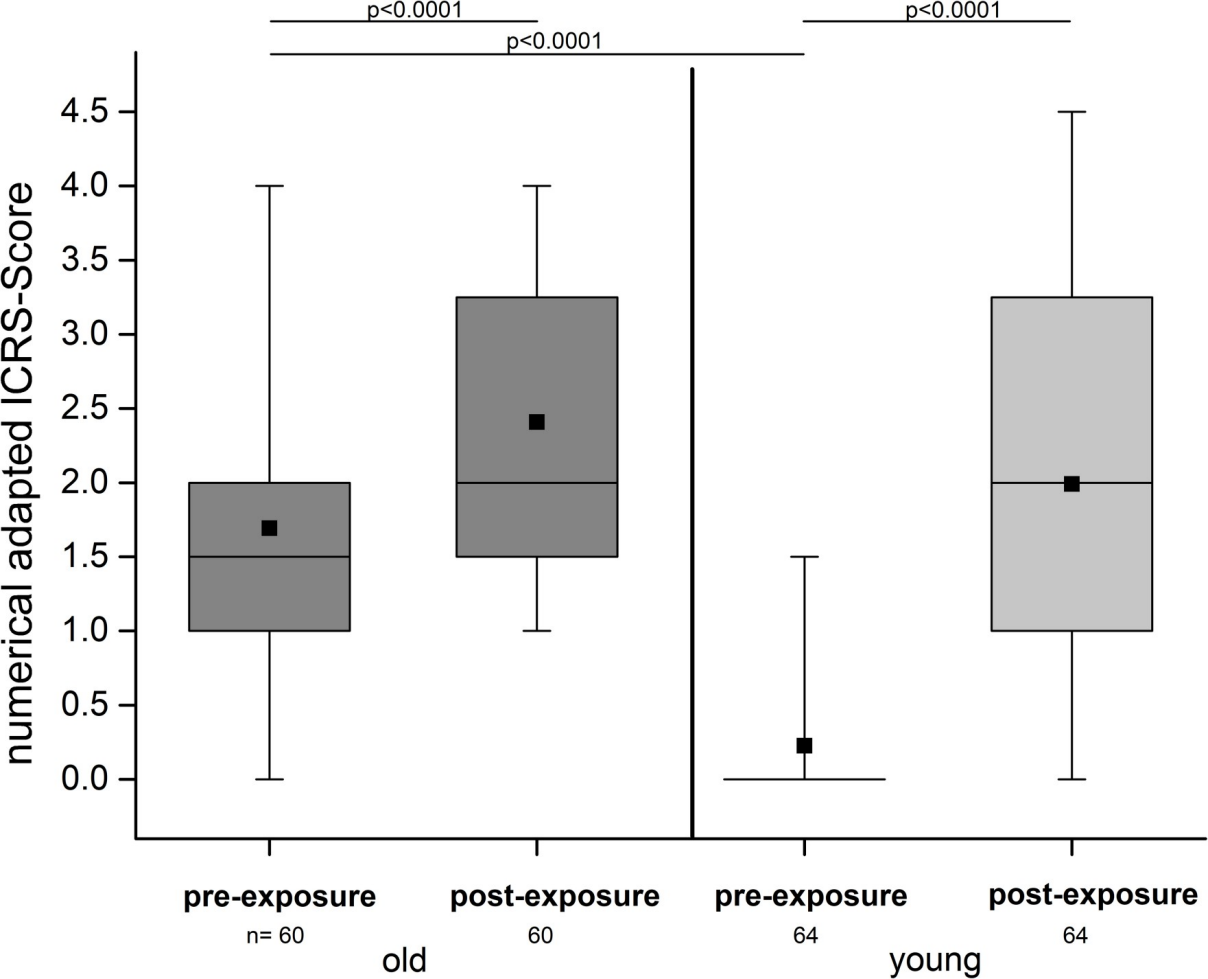
Numerically adapted ICRS-Score values before and after tribological exposure. On the left, the old group (dark grey) revealed medium score values of OA in the pre-exposure situation which were significantly increased after tribological exposure. The young group (light grey) on the right started with hardly any visible alterations and revealed significantly higher values after the exposure, similar to the conditions of the cartilage of the old animals after exposure (p = 0.0992).

We observed a statistically significant difference regarding the alterations on the cartilage surfaces (Fig 4) between the two groups with *p* < 0.0001, using the ICRS-System. In the old joints, we found a score value of 1.69 (±0.77) and in the young group a score value of 0.23 (± 0.44) in the mean (Table 2).

[In this and the following descriptive statistical figures the whiskers of the box and whiskers plots represent the minima and maxima, the boxes range from the 25% (bottom) to 75% (top) quartiles, the lines in the boxes illustrate the median, and the mean is represented by the square.]

#### Microscopic

**Fig 5:**
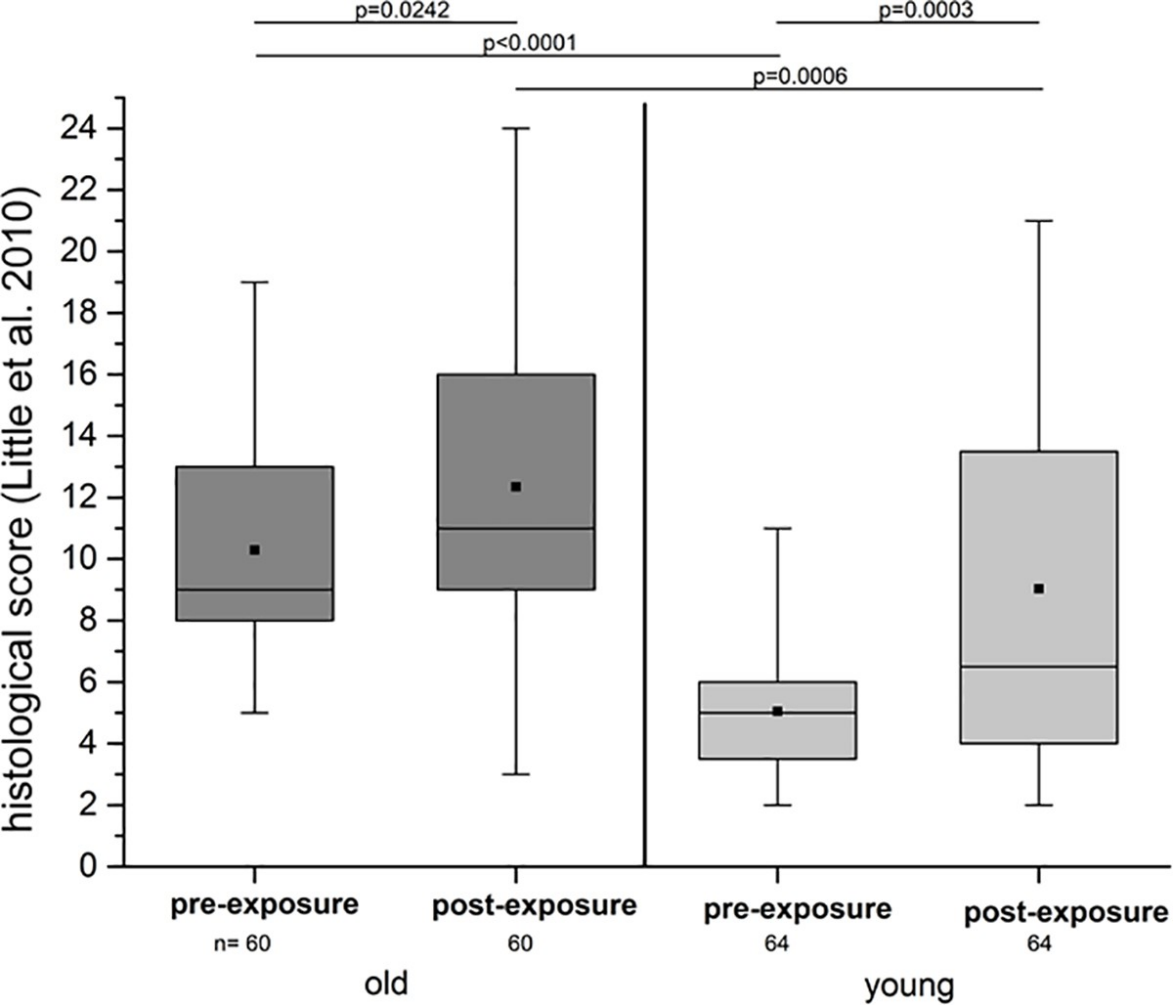
Little et al. [51] scoring of both groups before and after tribological exposure. Pre-exposure, the old group (left side; dark grey) showed medium signs of OA and was significantly distinguishable from the young group where only slight alterations were noticeable. After the tribological exposure, young and old specimens showed significant differences compared to their condition pre-exposure. So did the cartilage layers of the stifle joints of the young pigs (right side; light grey).

Cartilage specimens of the old group comprising 15 stifle joints showed degenerative changes with 10.28 (± 3.38) scoring points according to the Little et al. score [51] (Fig 5). This was different (*p* < 0.0001) from the young group consisting of 16 stifle joints, where 5.05 (± 2.12) scoring points were measured (Table 2).

The cartilage height of the specimens taken from the old pigs ranged from 0.9 mm (± 0.25 mm) (pin lateral) to 1.36 mm (± 0.59 mm) (pin medial) before the tribological exposure (Table 2).

The cartilage height of the specimens taken from the young pigs ranged from 0.88 mm (± 0.24 mm) (plate lateral) to 1.79 mm (± 0.47 mm) (pin medial) before the tribological exposure (Table 2).

### HL measurements, friction forces and cartilage assessment after tribological exposure

#### HL in the tribometer

Eight tribological pair—specimens had to be excluded from the evaluation due to technical failures that led to missing values in the group of cartilage taken from the young animals.

We detected an HL value (- 0.15 mm) that implies a height increase in the medial compartment of a joint of an old animal. We excluded this value from the evaluations.

**Fig 6:**
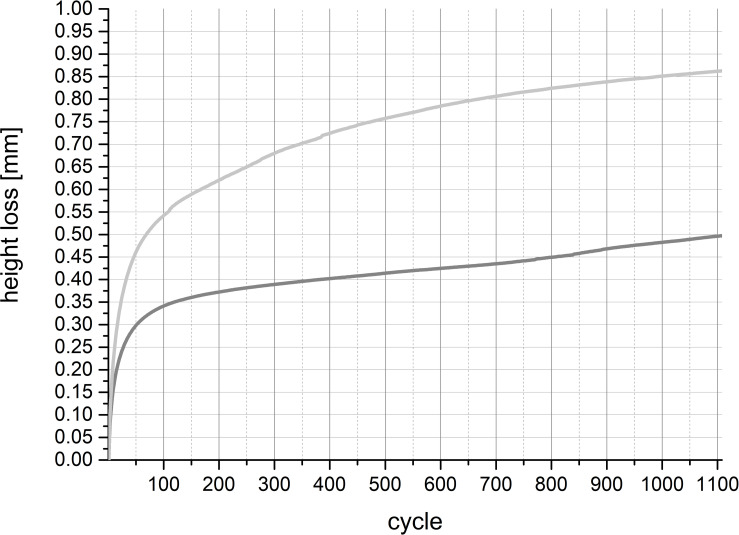
HL of the cartilage specimens during a 2.05 h tribological test with 1108 cycles. The averaged values of all specimens of the old group are depicted in dark grey, and those of the young group in light grey. The final value of the HL after tribological exposure was 0.52 mm for the old and 0.86 mm for the young group. The difference was significant (p < 0.0001). Standard deviations are reported in Table 2.

**Fig 7:**
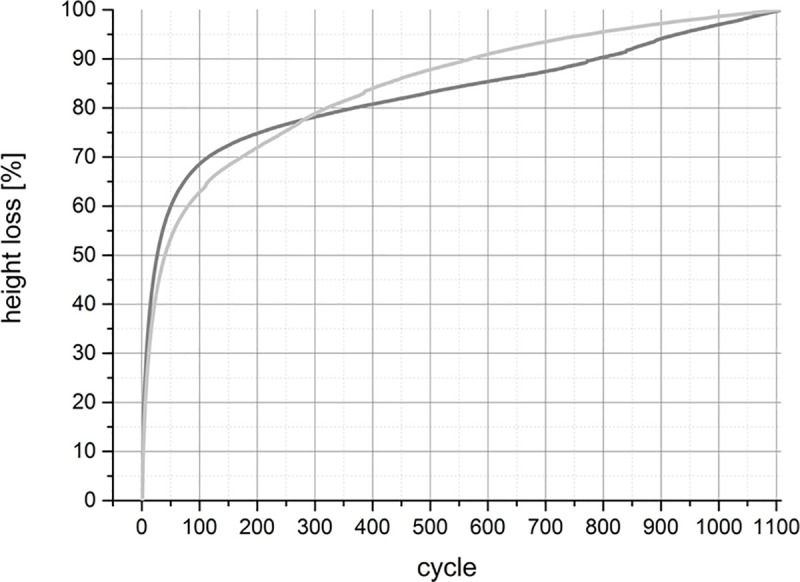
A comparison of the proportional HL during tribological exposure between the old group in dark grey and the young group in light grey averaged over all specimens. The specimens originating from old animals lost their proportional height a little more rapidly. However, the total loss of cartilage was lower (Fig 6). Throughout the first 100 cycles, both groups lost a major part of their initial height. After 100 cycles, the slope’s gradients indicating the rapidity of height decrease.

**Fig 8:**
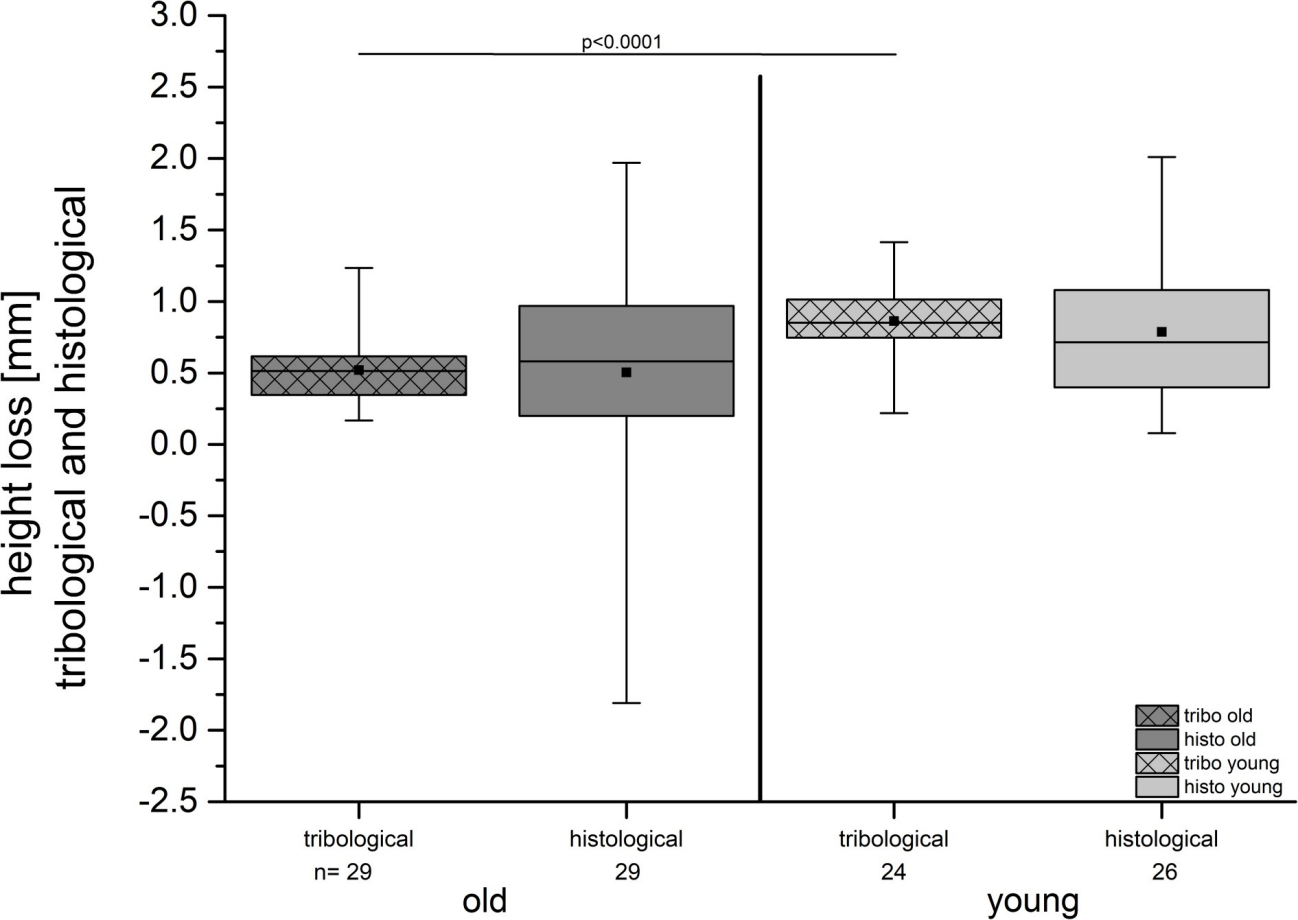
HL of the pair—specimens after tribological exposure was noticed in both groups (old and young); this was confirmed by the histological measurements. The HL was pronounced in the young cartilage specimens in comparison with the old cartilage. This result was significant in the tribological measurements. The histologically determined difference was not significant (p = 0.133).

During the tribological exposure we observed a continuous decrease in height of the pair—specimens in both groups (Figs 6–8).

The HL in the young group was pronounced and turned out to be significantly higher (*p* < 0.0001) than in the old group. The discrepancy between the two groups reached 0.34 mm after 1108 cycles (Fig 6, Table 2). In both groups, the highest progression in HL occurred throughout the first 100 cycles, where the HL was approximately 60% to 70% of their proportional value of height decrease (Fig 7). During the first 300 cycles the pair—specimens of old animals lost height more rapidly. This was reversed after cycle 300, at a point where proportional loss of height of the cartilage of young animals surpassed that of the cartilage of old animals, merging at 1100 cycles (Fig 7).

#### Friction force

We were able to assess the friction forces F_x_ acting on the pair–specimens taken from 28 compartments of the old animals and 24 compartments of the young animals. In the group of the old animals, 2 medial compartments and in the group of the young animals, 5 medial and 3 lateral compartments, could not be analyzed.

The friction force F_x_ was 2.25 N ± 1.15 N in the mean for the old animals and 1.89 N ±1.45 N for the young animals (p = 0.3225) (Table2). We found a statistically significant difference between the old and the young animals regarding the friction force F_x_ between the medial (p = 0.0073) but not between the lateral compartments (p = 0.5891). The friction forces in the lateral compartments of the old animals were 2.12 N ± 1.34 N and 2.42 N ± 1.61 N in those of the young animals. The medial compartments of the old animals produced 2.4 N ± 0.92 N, and those of the young animals 1.26 ± 0.98 N.

**Fig 9:**
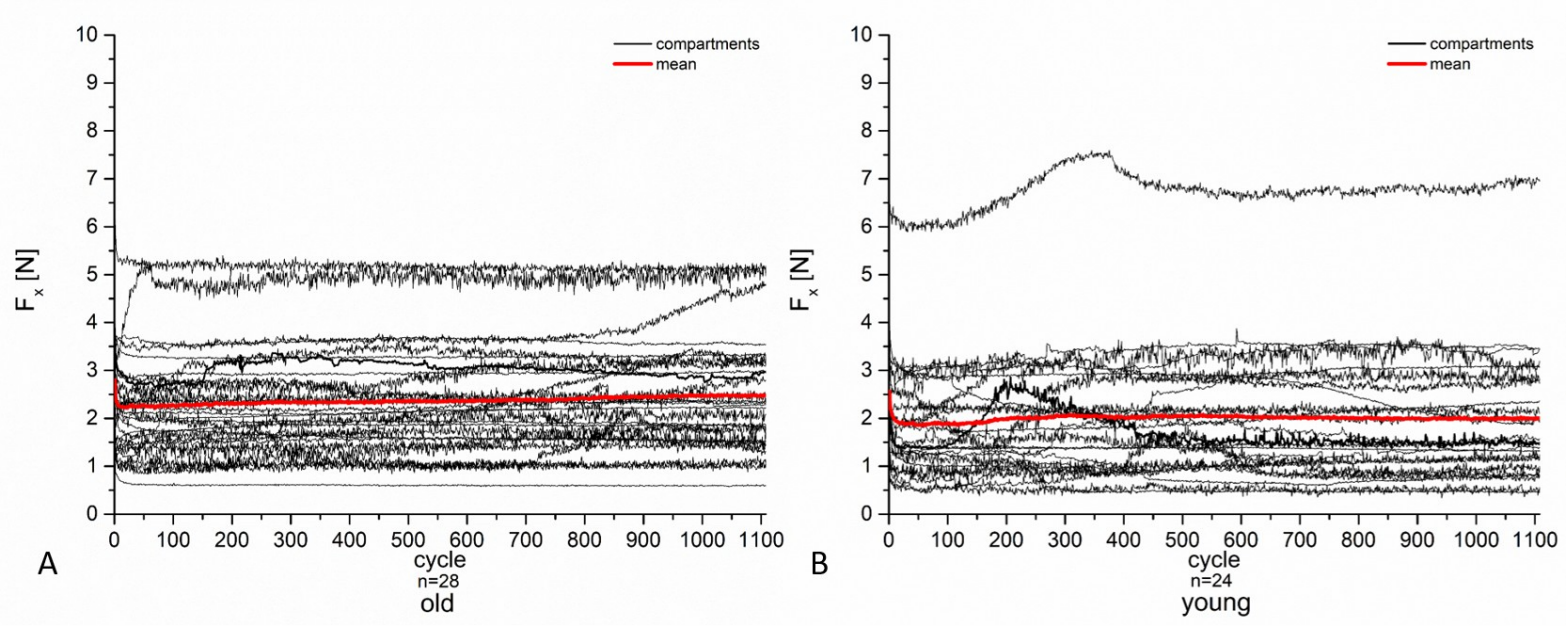
The figure represents the graphs of the friction forces of the cartilage of the old animals (A) and the young animals (B) over the entire running time. The red lines represent the mean curves of the compartments included in the tribological exposure in the respective group.

The friction forces revealed quite a linear and flat curve of progression in both groups over the entire running time (Fig 9).

The correlation coefficient for the relation between the HL and the friction force F_x_ was r = 0.32 (p = 0.0909) for the pair–specimens of the old animals and r = - 0.01 (p = 0.9540) for the pair–specimens the young animals. When comparing the compartments, we found no significant correlation between the two groups, with p–values ranging from p = 0.1385 to 0.9536.

#### Vertical load measurement

A comparison between the groups showed that both experienced a comparable amount of vertical pressure during testing as there was no statistical difference (p = 0.6441).

In the group of the old animals a mean load was calculated as 25.80 N (± 2.65 N) and in the group of the young animals it was 25.6 N (± 1.57 N) in the mean.

#### Macroscopic

**Fig 10:**
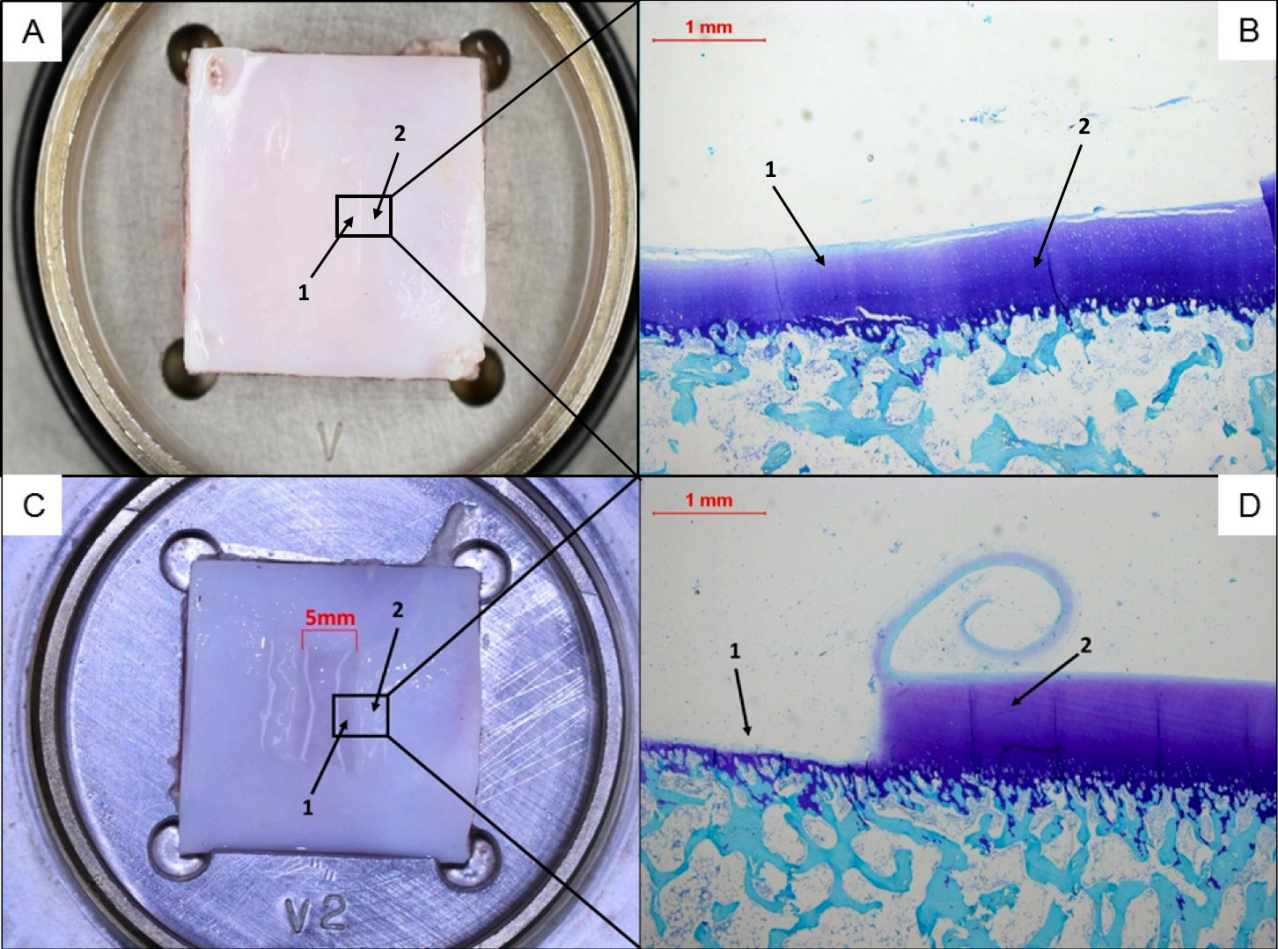
Representative images of the cartilage surface from a plate specimen of an old pig after tribological exposure, macroscopically (A) and histologically (B) in the cross section of the area where the pin moved. (1) indicates the track of the pin and (2) the control area. On slides (C) and (D) the same situation is shown for the plate specimen of a young pig. Here the track of the femoral pin with a diameter of 5 mm is clearly visible (1). The area directly next to it (2) served as the control area; this was not affected by the tribological exposure. (B) and (D) show the slides stained with toluidine blue. The superficial layer of the cartilage of the young pig was peeled off in a spiral shape (D).

When we looked at the condition of the cartilage before and after the experiment, we noted that the tribological exposure caused visible damage and led to a significant alteration of the cartilage surface in both groups (p < 0.0001). The alteration manifested itself in the form of prominent cartilage wear tracks on the plate, especially in the young group (Fig 10). Contrary to the pre-exposure evaluation, there were no significant differences between young and old in the post—exposure situation (p = 0.0992) (Fig 4, Table 2). The difference in the ICRS-score between the pre- and post-exposure situation was higher in the young group.

#### Microscopic

The tribological exposure had a significant impact on the cartilage in both groups, as the histological scores revealed higher values after the exposure than before (Fig 5).

The score value of the old group reached a mean value of 12.35 (± 5.06) and the young group a mean value of 9.03 (± 5.68) (Fig 5, Table 2).

#### Microscopic assessments of the HL of the cartilage

In all groups, a lower cartilage height for both the pins and the plates (Table 2) was measured after the tribological exposure compared to the initial height.

The height of the cartilage layers ranged from 0.86 mm (± 0.54 mm) (plate lateral) to 1.01 mm (± 0.55 mm) (pin medial) in the old group after exposure (Table 2).

In the young group, the height of the cartilage ranged from 0.37 mm (± 0.13 mm) (plate lateral) to 1.28 mm (± 0.4 mm) (pin medial) after exposure (Table 2).

In the mean, about 20% HL was detected in the pins and plates taken from the old pigs and nearly 37% HL in those taken from the young pigs.

The highest articular HL in the group of the old pigs was noted at the medial plate with nearly 33% HL after tribological exposure.

The HL after tribological exposure was more noticeable in the group of the young pigs. The lateral plate in particular showed the highest HL of nearly 58% in comparison with the state before tribological exposure.

#### Microscopic assessment of heights and HL of the compartments

**Fig 11:**
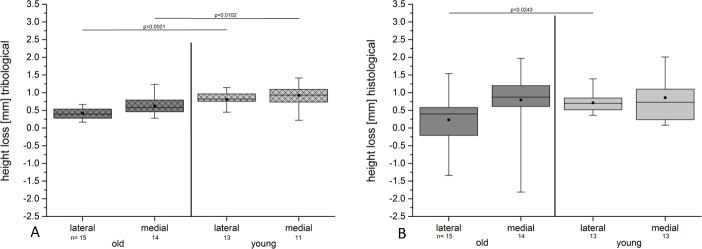
The results show a pronounced HL in the medial compartments both for the tribological and histological measurements in the old and in the young group. (A) The tribological assessment revealed significantly higher HL for the lateral and the medial compartments depending on the age of the animals. (B) The histological assessment showed a significantly higher HL only for the lateral compartment of the young group compared with the old group. Note the wider spread of data of the histological measurements compared to that of the tribological results.

We calculated the height of the cartilages taken from 15 lateral and 14 medial compartments of the old pigs and those taken from 13 lateral and 13 medial compartments of the young pigs (Fig 11, Table 2).

The HL of specimens from all compartments of the old pigs was in the mean 0.50 mm (± 0.82) mm and for the young pigs it was 0.79 (± 0.53 mm). The difference was not significant (p = 0.133).

The HL was significantly (p = 0.0243) lower for the lateral compartments of the old group (0.23 mm ± 0.69 mm) in comparison with the lateral compartments of the young group (0.71 mm ± 0.3 mm). For the medial compartments it was not significantly different (p = 0.8331).

#### Comparison of the tribological and the histologic HL

For the tribological measurements we identified 0.52 mm (± 0.23 mm) HL in the old group and 0.86 mm (± 0.26 mm) in the young group, taking all assessed specimens in each group into account (Fig 8, Table 2).

The histological measurements revealed a HL of 0.50 mm (±0.82 mm) for the old group and 0.79 mm (± 0.53 mm) for the young group (Fig 8, Table 2):

The difference in the HL of the old and of the young group was significant with respect to the tribological (p < 0.0001) but not the histological measurements (p = 0.133) (Fig 8).

The difference of the HL between the tribological and the histological measurements of the HL was 0.02 mm in the old group and 0.07 mm in the young group.

Looking at the different compartments, the HL was pronounced in the young group for the tribological and the histological assessments (Fig 11). The differences in the tribological measurements were significant in the medial and the lateral compartments for both the old and the young group (Fig 11). The histological assessment revealed significance only for the lateral site, not for the medial compartment (Fig 11).

#### Stratification of the cartilage from old animals regarding OA

To both, the OA + and in the OA–groups we were able to allocate 10 compartments each.

In the OA + group we found 7 medial compartments and 3 lateral compartments with a score value ≥ 11.5 points.

In the OA–group we found 3 medial and 7 lateral compartments with score values ≤ 8.5 points.

In the OA + group, the HL was 0.6 mm (± 0.29 mm) in the mean and ranged from 0.24 mm to 1.24 (median: 0.54 mm). In the OA–group the HL was 0.46 mm (± 0.15 mm) in the mean and ranged from 0.17 mm to 0.67 mm (median: 0.47 mm).

The difference between the OA+ and the OA- groups was not significant (p = 0.1938).

## Discussion

In the present study we included articular cartilage originating from old pigs, assuming that they had spontaneously developed OA in the stifle joints [34, 36]. Thus, we used the same origin as described by Kreinest et al. to harvest our samples [36]. If degenerative changes were also found in the articular cartilage, the animal model might become comparable to primary OA in humans [34]. The results of three different types of analyses, radiographic, macro- and microscopically, proved that the old pigs suffered from OA. Thus, we were then able to examine if degenerative changes of the articular cartilage could have an influence on the degree of damage after applying mechanical stress in form of tribological exposure.

Surface damages that can be expected in OA degenerated articular cartilage will increase the friction coefficient as reported by [45, 46], resulting in a higher shear force that stresses the tissue. Thus, we assumed that a tribological exposure could represent a powerful stress that would allow us to investigate biomechanically relevant characteristics of articular cartilage [43] expressed as HL of the cartilage layer.

The shear stress was to be quantified by measuring the friction forces acting at the surfaces of the tribological pair–specimens as F_x_, delivered by the force sensor in N.

A loss of the height of the articular cartilage layers *in vivo* causes the “narrowing of joint space”, which is a common parameter used worldwide in radiological OA diagnostic and follow-up, according to Kellgren and Lawrence [11, 12], as it radiologically reflects the wear and tear of the articular layers in a diarthrodial joint.

### OA-findings

The first hypothesis of the present study was that, in terms of OA-alterations, a statistically significant difference between stifle joints from old and young pigs could be revealed. Joints obtained from 5-year-old pigs showed clear signs of OA-changes in all assessment techniques, radiological-, macroscopical- or histological (Table 2).

The radiological assessment of the joints of old animals showed on average a medium grade of degeneration. None of these joints revealed the maximum grade IV of the Kellgren and Lawrence scoring system [11], which implies a complete narrowing of the joint space. In contrast, joints of young animals showed hardly any signs of radiological OA-alterations, with nearly 90% of the joints revealing grade 0 on the Kellgren and Lawrence grading scale [11]. Nevertheless, we are aware of certain limitations associated with the x-ray visualization of OA using the Kellgren and Lawrence grading system [11]. The standard for imaging OA in animals [35, 53] lacks sensitivity to early grade OA and allows only limited visualization of the grade [14, 54]. This might have led to the high amount of grade 0 in the joints of young pigs. Another limitation of the application of the x—ray score is the aspect of joint space narrowing. A view of the joints in a standing position could not be realized due to the animal—and cadaveric- origin of the stifle joints; thus, the examination of the joint space as originally described by Kellgren and Lawrence [11] was not possible. This might have led to missing values in the radiological assessment at the end of the scale of grading (IV) of OA.

For the macroscopic evaluation of the cartilage we chose the International Cartilage Repair Society (ICRS) classification designed by Brittberg and Peterson [15], as it is commonly used in animal models of OA [14, 55]. This assessment revealed various grades of OA in the joints of old animals and to a minor extent also in the joints from young animals (Figs 2 and 4, Table 2). The spectrum in the former group ranged from the complete absence of degenerative characteristics to a completely vanished cartilage layer, exposing the underlying subchondral bone (Fig 2). In addition, the medial compartments showed higher grades of OA than the lateral compartments (Table 2), which is in accordance with the observations of pronounced degenerative changes of medial compartments in animal models as described by Bendele and Hulman [56–58]. In contrast, the young group showed hardly any signs of degeneration. Therefore, a significant distinction between both groups in terms of OA could also be made when using macroscopic assessments.

In animal models, histological analysis is the gold standard [53, 59–61] for a proper assessment of the characteristic changes of the matrix and cells associated with the progression of OA. Several studies used the Mankin-score [16] or adapted versions thereof [62–64]. However, a follow- up initiative devised species-specific consensus scoring systems which are easily applicable and can be readily adopted [51, 65–70]. Considering that there was no specific score available for pigs, we used the OARSI scoring system of Little et al. [51] for goats and sheep as they are supposed to be comparable to pigs [35, 71]. In the present study, the analysis showed large variations of the grade of degenerations in the old group, ranging from slight surface irregularities to severe fibrillation or erosions (Fig 5). The old group presented clear signs of OA which were significantly dissimilar from the signs of OA evident in the specimens from young animals. In contrast, the latter was characterized by a smaller range of degeneration in the Little et al. scoring system [51] and showed, if any, only slight degenerative processes (Fig 5, Table 2).

In the present study, the gender of the animals of the young group is not known. But we can assume that the animals of the old group were female because they had been used for breeding. The missing information on the gender of the young animals might be a disadvantage of the used model, but they were young, and changes of the cartilage tissue in terms of degenerative changes were not likely. In addition, the availability of the specimens and the non-existent ethical concerns makes this animal model attractive.

Analyses of gene expression on menisci harvested from stifle joints of pigs obtained from the same slaughterhouse revealed higher grades of degeneration in old animals compared to those isolated from young stifle joints [36]. Based on these findings and as the analysis can also fail [35], we decided to forego gene expression, which is frequently used in OA research [35, 72, 73]. Instead, we focused our efforts on the biomechanical behavior. However, further studies may reveal that the OA of old pigs could be reflected at a gene expression level.

### Histological height measurements of the articular cartilage

The height of the cartilage layers of the pins and plates were measured histologically, representing the situation before and after tribological exposure. The heights were measured on the pictures of the histological slides of the osteochondral specimens. One has to keep in mind that the accuracy of this measurement depends largely on the quality of the slides and the placement of the cut. The true height of the cartilage layer can only be measured when the cut runs exactly perpendicular to the surface of the cartilage. A cut that does not run perpendicular to the surface of the layer leads to a higher estimation of the height than is truly the case. Because of the histological technique we applied and the geometrical circumstances it was impossible to measure a height lower than the true height. Thus, we have to point out that the cartilage heights will either be measured correctly or higher but not lower than the true height. Broad variances in the measured values could be explained by these facts (Table 2). The height of the cartilages could not be measured on 7 slides, 1 from the old and 6 from the young pigs because of defective histological slides.

When we looked at the height of the articular cartilage layer in the young group before the tribological exposure, we found quite similar conditions to those described by Fermor et al. of 6 months old pigs [74]. Fermor et al. reported that the medial femoral cartilage was the thickest (2.23 mm) and higher than the cartilage of the lateral condyle (2.08 mm) [74]. They described the tibial cartilage as being somewhat higher at the medial site (0.87 mm) than at the lateral site (0.82 mm) [74].

In the present study, the comparison between the old and the young group regarding the height of the cartilages before tribological exposure revealed lower cartilage heights of the pins in the group of the old pigs than in those in the group of the young. The opposite was true for the cartilage height of the plates (Table 2). Fermor et al. reported comparable results for old sheep, where the cartilage thickness of the condyle was higher than that of the tibia plateaus of young (8–12 months) sheep [74].

### Comparison of the HL, tribological and histological

In one case we found a “height increase” of the height of 0.15 mm of the pair—specimens in the tribometer measurements after tribological exposure. We could not find an explanation for this paradoxical result and we decided to exclude this value from any further evaluations. The non–exclusion of that value would have increased the difference in HL between the old and the young group. In addition, the histological height analysis of this compartment revealed an HL of 0.65 mm after tribological exposure. Thus, we assume that the exclusion of this value was justified in order to arrive at a sound evaluation. The significant difference between the old and the young group was not affected by this procedure.

The comparison of the tribologically and the histologically measured HL was possible when data of pair—specimens consisting of a femoral pin and the corresponding tibial plate were existent for both types of analysis. Thus, for the tribological analyses we got n = 29 datasets from the old pigs, and n = 24 from the young pigs (Table 2). For the histological analysis we got n = 29 datasets from the old pigs and n = 26 specimens from the young pigs (Table 2). Different sample sizes of the histological dataset resulted from lack of valid pair—specimens (see above).

The results of both the tribological and the histological measurements revealed the same trend: the HL tended to be higher in the young group than in the old group. Thus, the different behavior of the cartilage tissue of old and young pigs seemed to be validated, even if the difference was not significant for the histological but it was for the tribological data (Fig 8). The tribological height measurements of both cartilage layers (pin and plate respectively) were taken after the pin was pressed onto the surface of the plate with a pressure of about 1 MPa. In the histological slides, the heights of the cartilage layers were measured in an unloaded condition. There is a possibility that the settlement events of the osteochondral specimens and / or the sample holders could possibly compromise the HL measurements in the tribometer. However, one can assume that settlement events, if they did exist, were eliminated by the pre- compression of 1 MPa that was set. Thus, the histological and the tribological data were comparable and seemed to reflect the true HLs.

As the osteochondral specimens were fixed in formalin immediately after the tribological exposure we were not able to address the question if the HL was permanent or if the cartilage tissue could recover after a while as Katta et al. reported [5]. Looking at the levels of damage to the specimens that we saw after tribological exposure, we assumed that the HLs were permanent (Fig 10). We also noticed that the main HL occurred in the first 100 to 300 cycles. We measured the final HL after more than 1000 cycles (Figs 6 and 7). Thus, we think that the identified HLs were permanent and the tissue lost the capacity to recover. But we do not know if the HL was irreversible or if the tissue would be able to retain its elastic capacity to recover, and if so, after how many cycles and what period of time. This could be a topic for future studies.

After a long-term friction analyses (over 7 h) under loading conditions of up to 3.15 MPa, Katta et al. found lower articulation tracks on natural cartilage samples (0.087 mm– 0.152 mm) in the plate than in the GAG deficient samples (0.216 mm– 0.324 mm) [5]. Their values are comparable with the HL found in the plates of the old pigs with 0.2 mm in the present study, but hardly with HL in the young specimens (0.51 mm) (Table 2). We can assume that our test conditions were quite rigorous, leading to a fatigue of the tissue of the specimens. This is interesting, as Katta et al. [5] ran their tests for a much longer period of time compared to our study (7 h compared to our 2 h study). We used pins with a diameter of 5 mm in contrast to their 9 mm pins [5]. Thus, our test conditions were more demanding than those used by Katta et al. [5]. Ours lead to a measurable fatigue effect after a shorter period of time due to the smaller diameter of our pin specimen. However, the HL was always highly noticeable in the young group over the total running time (Fig 8).

### Reasons for different HLs

The results of the tribological exposure supported our second hypothesis, namely that there could be a difference in the HL between healthy and OA-affected cartilage samples during mechanical loading.

We found a significantly lower HL in OA cartilage compared to the HL in healthy cartilage (Fig 8, Table 2). However, one should be careful to attribute the results exclusively to the wear of cartilage tissue. Different viscoelastic behavior of the cartilage tissues [2, 75, 76] could also result in different measurements of the height of the pair—specimens. Especially the cartilage layer of the pin could have been highly affected as it was continuously under stress over the entire running time of approximately 2 hours. Pin and plate were under a different kind of stress as the latter was loaded in a dynamic and intermittent manner due to the reciprocating cyclic movement of the pin. Thus, different stress behavior could be expected for both, as the static loading may lead to a higher stress than a dynamic loading; Kääb et al. reported a decrease of the thickness of the cartilage of rabbits to 54% of the thickness of the controls under static loads, and to 78% under cyclic loading [77].

The histological data of the present study revealed the highest HL at both tibial plateaus, medial and lateral of the young animals and also at the medial tibia site of the old pigs (Table 2). But taking into account that old and degenerated cartilage has a higher stiffness than young cartilage [74, 78], we came to the assumption that where the cartilage of old animals is concerned, less wear and tear could be responsible for a lower HL. The highest HLs were found at the tibial sites of the young animals, lateral (57.95%) and medial (39.8%). Thus, one can assume that the quality of the articular cartilage of the young animals is different to that of the old animals, especially at the tibial sites. Fermor et al. reported that the equilibrium elastic modulus of young sheep at the tibial site is the lowest in comparison to the femoral site and in comparison to that of the cartilage of the old sheep tibial and femoral respectively [74].

Moore and Burris reported that the tibial cartilage of mature bovine stifle joints was of poorer quality and showed poorer tribological properties compared with that of the femoral cartilage [79].

Another indication for the assumption that the HL would be higher in the young group, was the fact that the cartilage layer from that group was higher before tribological exposure than that of older animals [74]. However, the differences were not very noticeable in the present study (Table 2).

### Friction forces

The friction occurring between both surfaces of the cartilage pair–specimen, was to be assessed as the friction force which is responsible for the shear stress acting on both surfaces.

The influence of friction specified as coefficient of friction (COF) and its potential role in maintaining the joint function and thus the cartilage layer has been extensively described in literature [29, 80–83]. Studies have shown that the COF might influence the wear and tear of the tissue during mechanical stress on cartilage [17, 84–87]. For the calculation of the COF, the friction force is a major component that acts parallel to the surfaces of the pair–specimens. Another component is the normal force acting perpendicular to the surfaces [88–90]. Thus, we wanted to explore if the different HLs could be caused, and thus explained, by different friction forces acting at the interface of the tribological surfaces of the articular pair—specimens.

Wong et al. [47] applied shear stress on human cartilage pairings under different conditions. They reported that degenerated cartilage revealed higher values in the “microscale shear testing” device than the normal cartilage, due to the higher roughness of the articular cartilage surface and a reduction of the shear modulus in the tissue [47]. They applied shear stress using a biaxial loading regime that consisted of compression and displacement of one osteochondral block against another [47]. In the present study, the loading conditions of the cartilage–pairing were comparable to those given by Wong et al. [47], as a biaxial loading regime was used in the same manner. However, we used a tribometer that was equipped with a degree of freedom to act in the vertical (Z -) direction. Thus, vertical displacements of the pin were detectable over the running time together with the friction force, as the force of resistance acting at the interface when the cartilages slid against each other.

Basalo and coworkers [10] reported on a similar approach when they tested the hypothesis that condroitinase ABC treated bovine articular cartilage would reveal a higher friction coefficient under creep conditions than untreated cartilage [10]. They found a creep of ε = 0.55 in both groups. The initial thickness of the specimens was 1.49 mm.

Their results are comparable with ours from the present study where microscopic HL assessments revealed a maximum of 33% HL in the group of old animals and 58% in the group of the young animals (Table 2). However, they did not detect a difference between the groups [10]. But one has to keep in mind that Basalo et al. [10] removed the deep zone of the cartilage tissue specimens before testing. In contrast to the present study where we analyzed the complete layer of the cartilage; the different procedure performed by Basalo et al. [10] could explain the discrepancy between their and our results regarding the significant HL between both groups (Table 2).

In the present study, the friction forces acting on the cartilage–specimens of the old animals were higher than the forces acting on the specimens of the young animals but not significantly so. Only one significant difference was noticed when comparing the specimens taken from the medial compartments of both groups (p = 0.0073), namely a lower F_x_ value measured in the young group (1.26 N vs. 2.4 N) than in the old group. But the HL in the medial compartment of the young animals yielded the highest value, both in the histological evaluation with 0.86 mm and in the tribometer measurement with 0.92 mm (Table 2).

Thus, the question arose if the HL and friction force could be interdependent variables. In the present study, we did not find any correlation between the mean value of HL and the mean value of the friction force F_x_ of the articular cartilage pair–specimens of the old and the young animals. The friction force was measured over the entire running time of the specimens, and we found quite constant curve progressions in both groups (Fig 9), so that substantial changes over the time of tribological exposure could be excluded. Thus, we assume that the friction acting on the surfaces is independent from the observed HL in the cartilage pair–specimens.

Caligaris et al. came to similar conclusions [80]. They found that the COF of human tibiofemoral cartilage does not increase with a higher grade OA [80]. Thus, it cannot be assumed that higher grades of OA automatically lead to higher wear. Based on these findings, it is reasonable to assume that, in the present study, the friction characteristic, measured as friction force, of OA cartilage and that of healthy cartilage had the same or no effect on the wear and could therefore be neglected.

In the present study, a drawback of the calculation of the friction force can be seen in the fact that the pin moved over an uneven plate and the friction force was not adjusted to the inclination of the surface at each contact point as described by Schütte et al. [90]. However, we think that the presented procedure is sound, because we calculated the friction force during each cycle, establishing the mean values of the friction force measured during the forward and the backward movements. This resembled the procedure described by Caligaris and Ateshian [6] for the calculation of the coefficient of friction of the articular cartilage of a bovine knee joint.

In the present study we were able to place the trajectories on comparable areas of the plates in all experiments as we had tried to identify the flattest possible regions of the plates observing the anatomical situation. We could not detect any noticeable differences in the unevenness of the tibial plate specimens between the old and the young animals. Each experiment was performed by the same experimenter (JPE) so that the personal bias was existent but constant in both groups. In addition, the normal loads in both groups were comparable (25.8 N vs. 25.6 N). The high sample size of specimens analyzed in the present study also enhances the validity of the findings.

The friction force seemed to be the better parameter to find out if the HL depended on the friction properties of the cartilage surface rather than the COF, as the latter must be calculated with additional measurements of the normal forces. The friction force reflects the force of resistance at the point where the pin moves over the plate. The wear phenomena that we noticed as partially deep tracks in the cartilage (Fig 10D) may have also have been caused by the pin acting as a kind of plough on the cartilage plate. If this was the case, the coefficient of friction could then not really be used to determine the wear profile. However, the curve progressions of the friction forces are quite flat and nearly homogenous in both groups (Fig 9). Thus, comparable friction behaviors seemed to have been existent in both groups, regardless of whether the cartilage layer was affected by osteoarthritic changes or damage during tribological exposure (Figs 5, 6 and 10D).

The observed HL seemed to be rather a fatigue behavior of the intrinsic biomechanical properties like moduli, creep etc. than due to the friction properties of the surfaces. HL and friction resistance of articular cartilage appeared to be independent variables in a tribological exposure test device as presented here.

The fact that friction forces are rarely mentioned in publications hampers a direct comparison with our findings. But the authors mostly described loading conditions when they calculated the COF. According to the formula given by Coulomb [88] and Czichos et al. [89] the friction force can be estimated when the COF and the normal force are given. Thus, looking at the results published by Bell et al. [45], it is possible to calculate friction forces ranging from 0.5 N to 7.25 N with COFs ranging from 0.02 to 0.29 for articular cartilage under a normal load of 25 N for different testing conditions [45]. Bell et al. [45] used a pin—on—plate friction simulator.

Northwood and Fisher [40] reported a friction value of 0.05 of bovine articular cartilage in a pin-on-plate friction and wear apparatus under a normal load of 30 N. According to Coulomb’s formula [88, 89] it is possible to calculate a friction force of 1.5 N.

Basalo et al. [10] calculated the friction coefficient μ_eq_ as 0.12 for the untreated cartilage as control and 0.19 for the cartilage that was treated with condroitinase ABC. Here they noted a significant increase in the friction coefficient. The friction coefficients given by Basalo et al. [10] corresponded to a friction force of 1.07 N and 1.69 N respectively, when applying the force–formula for the calculation of the friction coefficient [10, 88, 89]. These measurements of the friction forces are comparable with the results in the present study. But the results are not really comparable in terms of friction behavior over the observation times, as with Basalo et al. [10] the friction coefficient rises from 0.037 to 0.12 for the untreated cartilage and from 0.0053 to 0.19 for the treated tissue. Consequently, the friction forces increased, when a constant creep loading with 8.9 N was applied in both groups [10]. Whereas the friction forces in the present study remain almost constant over the complete observation times (Fig 9). We believe the different friction behavior is due to the fact that Basalo et al. [10] moved cartilage against glass and not against cartilage. It is well known that the value of the friction coefficient increases over the running time when cartilage is moved against glass but remains quite constant over longer periods of time when cartilage is moved against cartilage [28, 40, 45, 91], as was the case in the present study. Thus, the results of the present study are not really comparable to the results given by Basalo et al. [10]. However, the determination of the friction forces acting on articular cartilage reflects the physiological situation much better when cartilage is moved against cartilage, as was performed in the present study, rather than against an alloplastic material like glass.

However, the detected friction forces in the present study range from 1.26 N to 2.4 N and are comparable with results published in literature [10, 40, 45].

### Stratification of the cartilage from old animals regarding OA

The results indicating that OA—affected cartilage could be more resilient against tribological exposure are surprising, and it is possible that the age of the cartilage in particular is the reason for the lower HL.

Thus, we stratified the specimens according to the grade of the OA changes. By setting thresholds ≤ 8.5 and ≥ 11.5 scoring points, we created two balanced groups. That allowed us to show that old cartilage with less OA changes had less HL (0.46 mm) than cartilage with higher OA graded changes (0.6 mm). However, the difference was not significant. The disadvantage of that procedure was that there were no severe OA changes in the old group and no specimens without any degenerative changes either. We cannot exclude that the age of the cartilage alone could have had an impact on the stability of the cartilage. But on the other hand, there was no specimen in the old group that was completely free from degenerative changes. Thus, looking at the presented data, a higher age of the articular cartilage seems to inevitably go hand in hand with degenerative changes.

### The testing device and protocol

Pin and plate were harvested from the same compartment of a stifle joint of an animal, representing the cartilage layers as corresponding counterparts. We thus imitated the anatomical situation. But the combination of a pin and a plate simulating the sliding situation *in vitro* cannot entirely reproduce the physiological reality even if pin–on–plate devices were used in several former studies [5, 6, 10, 40, 45, 90, 91]. However, all cartilage pair–specimens were analyzed according to the same protocol where cartilage was slid against cartilage and in the same tribometer.

When we built the tribometer used in this study some years ago, we specifically designed it for the examination of cartilage–pair—specimens [22]. Based on the results of several experiments we were able to confirm the low COF of articular cartilage. We were also able to prove the applicability of the tribometer for the testing of tissue engineered constructs and for the detection of the influence of fetal calf serum (FCS) on artificially degenerated articular cartilage tissue with regard to the COF [22].

By doing so, the values of the friction forces are not distorted as is the case when using alloplastic materials like glass as a tribological partner of the articular cartilage [10].

The use of PBS as lubricant in the present study could have intensified the shear stress on the surface of the cartilage pair- specimens as described by Wong et al. [47]. However, considering the more rigorous examination conditions, we see the presented examination protocol as an advantage rather than a disadvantage as it could reveal weaknesses of the assessed tissue earlier. We believe that the use of PBS as lubricant has several advantages: preparation occurs under reproducible conditions in a laboratory, and handling and availability are both straightforward. Another advantage of PBS is the fact that it has frequently been used in tribological examinations of articular cartilage tissue before [10, 22, 92]. This makes our results more comparable. To our knowledge, there is no general agreement on the substance that should be used as a lubricant in tribological examinations of articular cartilage, but in our trials all specimens were examined with PBS as lubricant.

The mechanical stress on the specimens in the present study was certainly higher than *in vivo* even though we applied only 1 MPa which represents a low pressure that can occur *in vivo* [4]. However, one has to consider, that there is not only one physiological condition but a wide range of loading parameters in daily live [93, 94]. But both qualities of cartilage, the OA—degenerated from the old animals and the healthy from the young animals, were stressed following the same protocol.

We believe that we applied a testing protocol which stressed the cartilage more than would have been the case under physiological conditions but it could reveal weak points of cartilage specimens earlier.

We cannot give sufficient explanations for the observations made in the present study that old and degenerated cartilage may be more resilient than young and healthy cartilage. However, the results could give some cause for hope for patients suffering from OA, as the articular cartilage seemed to withstand mechanical stress better than healthy cartilage.

Further studies should give clarity.

## Conclusion

We were able to confirm that articular cartilage from old porcine tibio-femoral joints may serve as animal model for primary OA research, presenting a broad range of OA alterations. Stifle joints from young pigs that showed hardly any signs of degenerative alterations served as useful controls. The present study revealed a more resilient behavior of the OA affected cartilage under tribological shear stress exposure compared to the healthy cartilage from young pigs. Friction forces acting at the surface of the articular cartilage and HL seem to be independent parameters. The frictional capabilities of the articular cartilage seemed to remain unchanged over the assessed observation time independent of the condition of the cartilage tissue and the amount of wear and tear in terms of HL. The applied mechanical loading in a pin-on-plate device might stress the articular cartilage more than would be the case under physiological conditions thus revealing potentially existing weaknesses of the assessed tissue if existing.

## Supporting information

S1 Table Raw data excel file. The table provides the data of: the height of articular cartilage before and after tribological exposure measured histologically (sheet 1), the tribologically measured height loss (HL) (sheet 2), the friction forces (sheet 3) and the scoring results according to Little et al., the ICRS-score and Kellgren and Lawrence (x-ray) (sheet 4).
(XLSX)
